# Alternative prostate cancer grading systems incorporating percent pattern 4/5 (IQ-Gleason) and cribriform architecture (cGrade) improve prediction of outcome after radical prostatectomy

**DOI:** 10.1007/s00428-022-03301-y

**Published:** 2022-02-14

**Authors:** Neslisah Seyrek, Eva Hollemans, Eleni-Rosalina Andrinopoulou, Susanne Osanto, Rob C. M. Pelger, Henk G. van der Poel, Elise Bekers, Sebastiaan Remmers, Ivo G. Schoots, Geert J. L. H. van Leenders

**Affiliations:** 1 Department of Pathology, Erasmus MC Cancer Institute, University Medical Centre Rotterdam, P.O. Box 2040, 3000 CA Rotterdam, The Netherlands; 2 Department of Radiology & Nuclear Medicine, Erasmus MC Cancer Institute, University Medical Centre Rotterdam, Rotterdam, The Netherlands; 3 Department of Biostatistics, Erasmus MC Cancer Institute, University Medical Centre Rotterdam, Rotterdam, The Netherlands; 4 Department of Epidemiology, Erasmus MC Cancer Institute, University Medical Centre Rotterdam, Rotterdam, The Netherlands; 5 Department of Medical Oncology, Leiden University Medical Centre, Leiden, The Netherlands; 6 Department of Urology, Leiden University Medical Centre, Leiden, The Netherlands; 7 Department of Urology, Antoni Van Leeuwenhoek-Netherlands Cancer Institute, Amsterdam, The Netherlands; 8 Department of Pathology, Antoni Van Leeuwenhoek-Netherlands Cancer Institute, Amsterdam, The Netherlands; 9 Department of Urology, Erasmus MC Cancer Institute, University Medical Centre Rotterdam, Rotterdam, The Netherlands

**Keywords:** Prostate cancer, Radical prostatectomy, Gleason score, Grade group, Cribriform, Percent

## Abstract

Percentage Gleason pattern 4, invasive cribriform and/or intraductal carcinoma (IC/IDC) and minor pattern 5 are recognized as independent parameters for prostate cancer outcome, but are not incorporated in current grade groups (GGs). Two proof-of-principle studies have proposed alternative grading schemes based on percentage Gleason pattern 4/5 (integrated quantitative Gleason score; IQ-Gleason) and IC/IDC presence (cribriform grade; cGrade). Our objective was to compare the performance of GG, IQ-Gleason and cGrade for predicting biochemical recurrence and metastasis after radical prostatectomy (RP). RP specimens of 1064 patients were pathologically reviewed and graded according to the three schemes. Discriminative power for prediction of biochemical recurrence-free (BCRFS) and metastasis-free (MFS) survival was compared using Harrell’s *c*-index. The GG distribution at RP was 207 (19.4%) GG1, 472 (44.4%) GG2, 126 (11.8%) GG3, 140 (13.2%) GG4 and 119 (11.2%) GG5. Grading according to 5-tier IQ-Gleason and cGrade systems led to categorical shifts in 49.8% and 29.7% of cases, respectively. Continuous IQ-Gleason had the best performance for predicting BCRFS (*c*-index 0.743, 95% confidence interval (*CI*) 0.715–0.771), followed by cGrade (*c*-index 0.738, 95%*CI* 0.712–0.759), 5-tier categorical IQ-Gleason (*c*-index 0.723, 95%*CI* 0.695–0.750) and GG (*c*-index 0.718, 95%*CI* 0.691–0.744). Continuous IQ-Gleason (*c*-index 0.834, 95%*CI* 0.802–0.863) and cGrade (*c*-index 0.834, 95%*CI* 0.808–0.866) both had better predictive value for MFS than categorical IQ-Gleason (*c*-index 0.823, 95%*CI* 0.788–0.857) and GG (*c*-index 0.806, 95%*CI* 0.777–0.839). In conclusion, the performance of prostate cancer grading can be improved by alternative grading schemes incorporating percent Gleason pattern 4/5 and IC/IDC.

## Introduction

The Gleason grading system is the cornerstone of risk assessment and prediction of clinical outcome in prostate cancer (PCa) patients. In radical prostatectomy (RP) specimens, the Gleason score (GS) is determined by adding the two most frequent growth patterns resulting in a final score of 2 to 10. Based on the work of Pierorazio et al. and Epstein et al., the GS is categorized into five grade groups (GGs) [6, 13]. The International Society of Urological Pathology (ISUP), World Health Organization (WHO) and Genitourinary Pathology Society (GUPS) endorse reporting of GGs in conjunction with GS [4, 5, 9]. The advantages of the GG system are its simplicity, explicit distinction of GS 3 + 4 and 4 + 3 and categorization of GS ≤ 6 as GG1.

In recent years, the clinical relevance of pathological parameters such as Gleason pattern 4 quantity, presence of invasive cribriform and intraductal (IDC) carcinoma, and minor/tertiary Gleason patterns has been well acknowledged [1–3, 7, 8, 12, 15]. For instance, the risk of post-operative biochemical recurrence is increasing with incremental Gleason 4 pattern quantity and presence of minor Gleason pattern 5 [1, 2, 10, 15]. Furthermore, invasive cribriform and/or intraductal carcinoma (IC/IDC) has been associated with biochemical recurrence, metastatic disease and disease-specific survival [3, 7, 8, 12]. Consequently, the ISUP and GUPS both recommend including these specific features in pathology reports [4, 19].

Although of prognostic significance, it is unclear how percent pattern 4, IC/IDC and minor pattern 5 altogether translate to individual risk assessment and should be used in clinical practice. For instance, it is unknown whether patients with GG2 with 20% Gleason pattern 4, IDC and tertiary pattern 5 have worse outcome than those with GG3 with 60% pattern 4 but no cribriform carcinoma or tertiary pattern 5. Few groups have demonstrated that alternative grading schemes incorporating some of these pathological factors had significantly better discriminative value than current GGs [14, 17, 18]. On biopsy and RP specimens, Sauter et al. found that an integrated quantitative Gleason (IQ-Gleason) score, which is purely based on Gleason pattern 4 and 5 quantities, led to better risk stratification for biochemical recurrence-free survival (BCRFS) [14]. Alternatively, modification of the GG system for the presence of IC/IDC on biopsies — labelled as cribriform grade (cGrade) — resulted in improved discriminative value for disease-specific and metastasis-free survival (MFS) [18].

Although these proof-of-principle studies reveal that optimization of the current GG system is possible, no studies have independently validated the prognostic value of these models. The objective of the current study was to compare the discriminative ability of GG, IQ-Gleason and cGrade for BCRFS and MFS in a RP cohort.

## Materials and methods

### Patient selection

Patients who had undergone RP for prostatic adenocarcinoma at three medical centres in The Netherlands between 2000 and 2017 were included in this study; 854 patients were operated at Erasmus MC, University Medical Centre, Rotterdam; 96 at Leiden University Medical Centre (LUMC), Leiden; and 137 at Antoni van Leeuwenhoek Hospital, The Netherlands Cancer Institute (NKI), Amsterdam. While the RP specimens of Erasmus MC were unselected consecutive samples, those from LUMC and NKI were selected for having GG3–5 disease in their original pathology report to increase the number of high-grade tumours. Patients who had undergone hormonal, radiation and/or viral therapy (*n* = 23) before RP were excluded. RP specimens were fixed in neutral-buffered formalin, after which they were sectioned transversely and embedded entirely for diagnostic purposes. All slides were available for pathology review. The institutional Medical Research Ethics Committee approved this study (MEC-2018–1614).

### Pathological evaluation

All 1064 RP specimens were reviewed in joint sessions by two investigators (EH, GvL), blinded to clinical outcome. In case of discordances, the assessment of the senior genito-urinary pathologist (GvL) was included in the study database. For each specimen, the following features were recorded: GS and GG according to the 2014 ISUP/2016 WHO guidelines, pT stage according to the American Joint Committee on Cancer (AJCC) TNM 8th edition, surgical margin status, presence of IC/IDC and percent Gleason 4 and 5 growth patterns. Invasive cribriform and IDC were not distinguished and grouped for all analyses. In case of multifocality, we only monitored the characteristics of the index tumour defined as the tumour with the highest grade, stage or volume. Tertiary patterns occupying < 5% of the tumour volume and IDC were not included in the GG. The GG concordance rate at revision was 88/135 (65.2%) for RP from NKI and 39/94 (41.5%) for specimens from LUMC; this discordance rate was affected by the fact that the original tumour grading had been performed by a large number of general pathologists and that several samples were originally graded before the 2005 ISUP consensus meeting.

### Clinical follow-up

Clinical follow-up after RP consisted of 6 monthly and later annual monitoring of serum prostate-specific antigen (PSA) levels. Biochemical recurrence was defined as PSA levels ≥ 0.2 ng/ml measured at two consecutive points in time, at least 3 months apart with undetectable PSA levels after RP. Post-operative lymph node and distant metastases were confirmed by biopsy, imaging or multidisciplinary consensus.

### IQ-Gleason and cGrade assessment

The IQ-Gleason score is calculated by summing Gleason pattern 4 and 5 percentages. Ten points are added if any Gleason pattern 5 is present and 7.5 points extra if it exceeds 20%. This results in a continuous IQ-Gleason score from 0 to 117.5 points. For comparison purposes, we a priori categorized IQ-Gleason into five ordinal groups as follows: 0–25, 26–50, 51–75, 76–100 and 101–117.5.

**Fig. 1:**
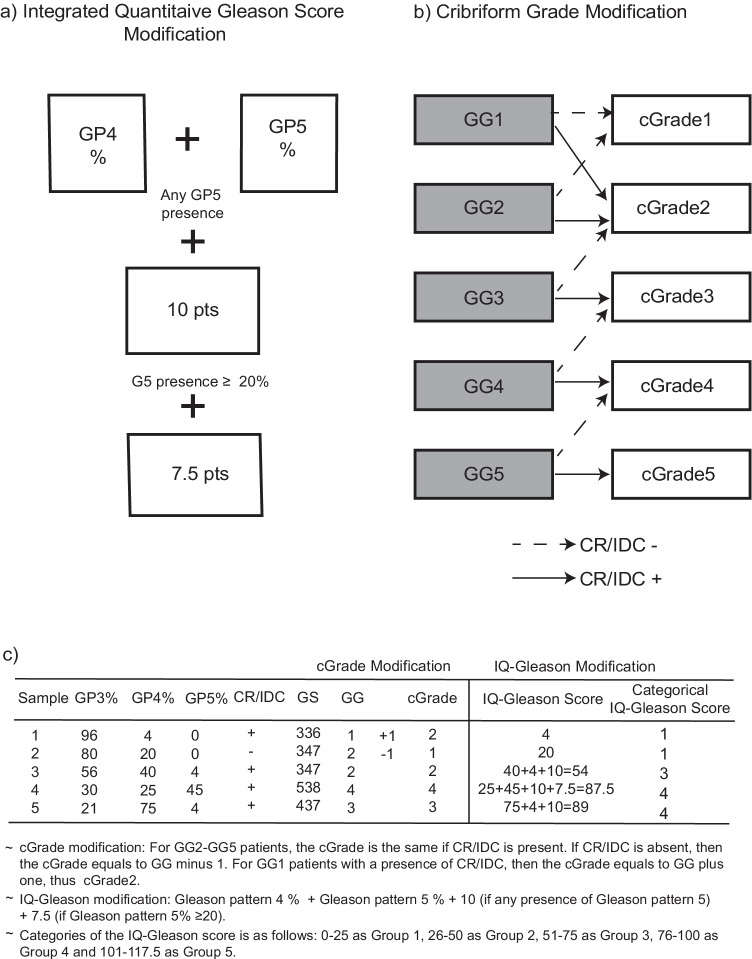
Overview of (**a**) integrated quantitative Gleason (IQ-Gleason), (**b**) cribriform grade (cGrade) and (**c**) examples of prostate cancer grading

cGrade is based on the GG system, where the grade is decreased by 1 point in case no invasive cribriform and intraductal carcinoma is present in GG2–5 tumours. In the rare case of GG1 with IDC, 1 point is added leading to cGrade2 classification. Schematic descriptions and examples of IQ-Gleason and cGrade are depicted in Fig. 1.

### Statistical analysis

Missing PSA values (*n* = 27) were imputed using the median PSA value. BCRFS and MFS were analysed using the Cox proportional hazards model and visualized by Kaplan–Meier curves. Hazard ratios (*HR*) for survival time were calculated using univariate Cox proportional hazard regression. For all models, Cox proportional hazard assumptions were met. Harrell’s concordance index (*c*-index) was used to quantify the discriminative ability of the grading models. Bootstrapping was used to obtain unbiased estimates of the model’s performance and 95% confidence interval (*CI*). Statistics were performed using SPSS version 25 (IBM, Chicago, IL, USA) and R version 4.0.4 (R, Vienna, Austria). Results were considered significant when the two-sided *p* value was < 0.05.

## Results

### Patient characteristics

The median age of the 1064 patients who had undergone RP was 64.6 years (interquartile range (*IQR*) 60.2–68.1), and the serum PSA level was 8.3 ng/ml (*IQR* 6.0–13.2). The GG distribution was as follows: 207 (19.4%) GG1, 472 (44.4%) GG2, 126 (11.8%) GG3, 140 (13.2%) GG4 and 119 (11.2%) GG5. The pathological tumour stage was pT2 in 582 (54.7%), pT3a in 334 (31.4%), pT3b in 145 (13.6%) and pT4 in 3 (0.3%) patients. Positive surgical margins were observed in 390 (36.7%) patients. Out of 665 (62.5%) patients who had undergone pelvic lymph node dissection, 63 (5.9%) had lymph node metastasis. IC/IDC was present in 568 (53.4%) patients, and 118 (11.1%) had tertiary (< 5%) Gleason pattern 5. The median clinical follow-up for men without events was 54.1 (*IQR* 12.2–95.2) months. Biochemical recurrence occurred in 342 (32.1%) patients after 36.3 months (*IQR* 9.3–78.5), and 136 (12.8%) men developed distant metastasis after 58.5 months (*IQR* 18.6–100.1 months). The clinical and pathological characteristics stratified per GG are shown in Table 1.

**Table 1:** Clinicopathological characteristics of patients undergoing RP (*n* = 1064) for prostate cancer

	GG1 *n* = 207	GG2 *n* = 472	GG3 *n* = 126	GG4 *n* = 140	GG5 *n* = 119
Age (*IQR*)	63.2 (59.8–66.7)	64.6 (59.9–68.0)	66.2 (60.9–69.9)	65.3 (61.4–68.5)	64.7 (60.8–70.0)
PSA (*IQR*)	6.3 (4.0–9.2)	8.3 (6.0–12.9)	11.6 (7.2–19.2)	10.0 (7.2–68.5)	11.3 (7.1–19.0)
pT					
T2	185 (89.4%)	268 (56.8%)	37 (29.4%)	67 (47.9%)	25 (21.0%)
T3a	20 (9.7%)	169 (35.8%)	53 (42.1%)	44 (31.4%)	48 (40.3%)
T3b	2 (1.0%)	35 (7.4%)	36 (28.6%)	28 (20.0%)	44 (37.0%)
T4	0 (0.0%)	0 (0.0%)	0 (0.0%)	1 (0.7%)	2 (1.7%)
Positive surgical margins	35 (16.9%)	156 (33.1%)	64 (50.8%)	67 (47.9%)	68 (57.1%)
Pelvic lymph node dissection	134 (64.7%)	262 (55.5%)	87 (69.0%)	91 (65.0%)	91 (76.5%)
Metastasis on pelvic lymph node dissection	0 (0.0%)	13 (2.8%)	21 (16.7%)	12 (8.6%)	17 (14.3%)
Tertiary Gleason 5 pattern	0 (0.0%)	56 (11.9%)	53 (42.1%)	9 (6.4%)	0 (0.0%)
Invasive cribriform and/or intraductal carcinoma	9 (4.3%)*	252 (53.4%)	118 (93.7%)	87 (62.1%)	102 (85.7%)
Biochemical recurrence	16 (7.7%)	107 (22.7%)	74 (58.7%)	68 (48.6%)	77 (64.7%)
Distant metastasis	0 (0.0%)	18 (3.8%)	35 (27.8%)	36 (25.7%)	47 (39.5%)

^*^Nine cases represent IDC

### IQ-Gleason and cGrade

The distribution of the IQ-Gleason categories was 497 (46.7%) IQ-Gleason1, 203 (19.1%) IQ-Gleason2, 117 (11.0%) IQ-Gleason3, 163 (15.3%) IQ-Gleason4 and 84 (7.9%) IQ-Gleason5. GG and IQ-Gleason categories were similar in 545 (51.2%) men (Table 2a). The most prominent difference was the categorization of 287 (60.8%) GG2 patients as IQ-Gleason1, which can be attributed to patients with < 25% Gleason pattern 4. Only 50/140 (35.7%) of GG4 patients were classified as IQ-Gleason4, which was mostly due to categorization of 71/76 (93.4%) GS 3 + 5 = 8 tumours in lower groups, while 62/126 (49.2%) GG3 tumours were upgraded and 57/119 (47.9%) GG5 downgraded.

**Table 2:** Distribution of grade groups (GGs), categorical integrated quantitative Gleason (IQ-Gleason) score and cribriform grade (cGrade)

(a)						
	IQ-Gleason1	IQ-Gleason2	IQ-Gleason3	IQ-Gleason4	IQ-Gleason5	Total
GG1	207	-	-	-	-	207
GG2	287	163	22	-	-	472
GG3	-	1	63	59	3	126
GG4	3	39	29	50	19	140
GG5	-	-	3	54	62	119
Total	497	203	117	163	84	1064
(b)						
	cGrade1	cGrade2	cGrade3	cGrade4	cGrade5	Total
GG1	198	9	-	-	-	207
GG2	219	253	-	-	-	472
GG3	-	8	118	-	-	126
GG4	-	-	52	88	-	140
GG5	-	-	-	17	102	119
Total	417	270	170	105	102	1064
(c)						
	IQ-Gleason1	IQ-Gleason2	IQ-Gleason3	IQ-Gleason4	IQ-Gleason5	Total
cGrade1	356	56	5	-	-	417
cGrade2	138	107	21	4	-	270
cGrade3	1	25	71	68	5	170
cGrade4	2	15	18	45	25	105
cGrade5	-	-	2	46	54	102
Total	497	203	117	163	84	1064

The cGrade distribution was 417 (39.2%) cGrade1, 270 (25.4%) cGrade2, 170 (16.0%) cGrade3, 105 (9.9%) cGrade4 and 102 (9.6%) cGrade5. In total, 759 (71.3%) men had similar GG and cGrade category (Table 2b). Incorporation of IC/IDC in cGrade had the most impact on GG2 patients, of whom 219 (46.4%) were categorized as cGrade1. Furthermore, 52/140 (37.1%) GG4 tumours were classified as cGrade3. cGrade and IQ-Gleason category were concordant in 633 (59.5%) men with considerable redistribution among all groups (Table 2c).

### Biochemical recurrence-free survival

**Fig. 2:**
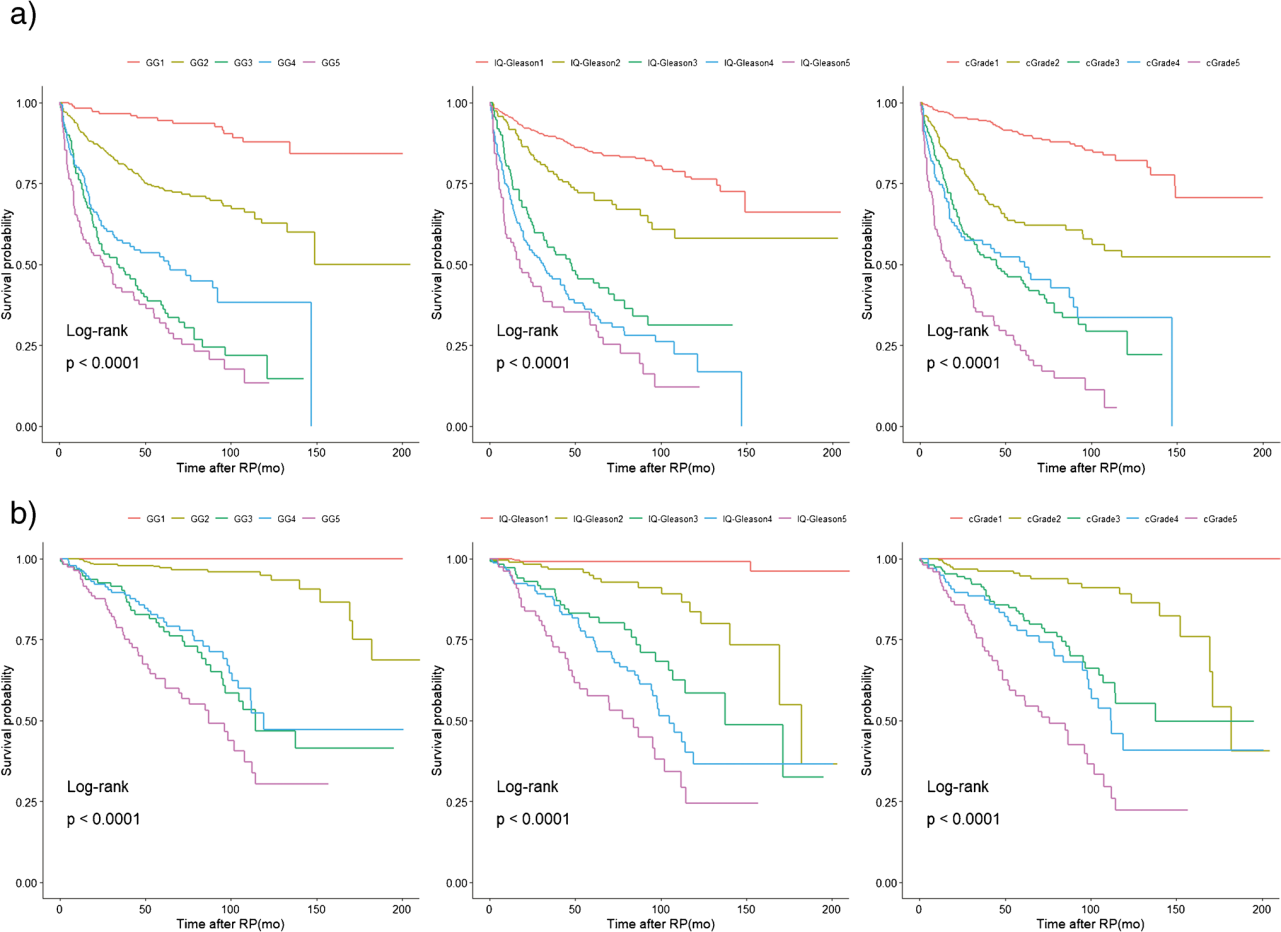
**a** Biochemical recurrence-free and **b** metastasis-free survival (log-rank) stratified by grade group (GG), categorical integrated quantitative Gleason (IQ-Gleason) and cribriform grade (cGrade)

**Fig. 3:**
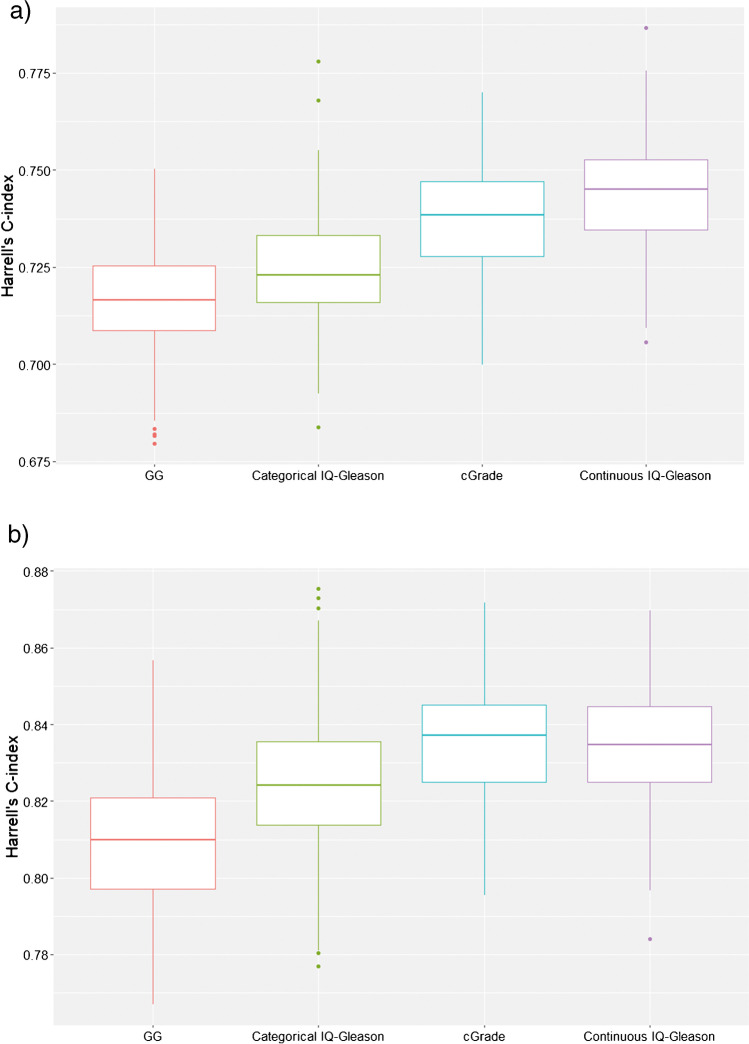
Harrell’s *c*-index for biochemical recurrence-free (**a**) and metastasis-free (**b**) survival of grade groups (GGs), categorical integrated quantitative Gleason (IQ-Gleason), cribriform grade (cGrade) and continuous IQ-Gleason

Post-operative biochemical recurrence occurred in 16 (7.7%) GG1, 107 (22.7%) GG2, 74 (58.7%) GG3, 68 (48.6%) GG4 and 77 (64.7%) GG5 tumours. For categorized IQ-Gleason, recurrence rates were 76/497 (15.3%), 50/203 (24.6%), 59/117 (50.4%), 98/163 (60.1%) and 59/84 (70.2%), respectively, and for cGrade 48/417 (11.5%), 77/270 (28.5%), 88/170 (51.8%), 54/105 (51.4%) and 75/102 (73.5%). BCRFS of the three grading models is shown in Table 3 and Fig. 2a. The cGrade model (*c*-index 0.738, 95% confidence interval (*CI*) 0.712–0.759) had higher discriminative power than conventional GGs (*c*-index 0.718, 95%*CI* 0.691–0.744). IQ-Gleason analysed as a continuous variable (*c*-index 0.743, 95%*CI* 0.715–0.771) had the highest discriminative ability for BCRFS, although this decreased considerably after its categorization (*c*-index 0.723, 95%*CI* 0.695–0.750) (Fig. 3a).

**Table 3:** Cox proportional hazard models for biochemical recurrence-free survival stratified by grade group (GG), categorical integrated quantified Gleason (IQ-Gleason) score and cribriform grade (cGrade)

Group	Grade group	IQ-Gleason	cGrade
	*HR (95%CI)*	*HR (95%CI)*	*HR (95%CI)*
1	*ref*	*ref*	*ref*
2	4.1 (2.4–7.0)	2.0 (1.4–2.8)	3.6 (2.5–5.2)
3	14.4 (8.3–24.9)	4.8 (3.4–6.8)	7.2 (5.1–10.3)
4	10.2 (5.9–17.6)	6.5 (4.8–8.8)	6.9 (4.6–10.1)
5	18.0 (10.4–31.0)	8.5 (6.0–12.0)	14.5 (9.5–19.9)

All *p*-values are < 0.001

*CI* confidence interval, *HR* hazard ratio, *ref* reference

### Metastasis-free survival

Post-operative distant metastases were observed in none of GG1, 18 (3.8%) GG2, 35 (27.8%) GG3, 36 (25.7%) GG4 and 47 (39.5%) GG5 tumours. According to 5-tier IQ-Gleason, 4/497 (0.8%), 17/203 (8.4%), 27/117 (23.1%), 51/163 (31.1%) and 37/84 (44.0%) patients experienced distant metastasis, respectively. For cGrade, metastatic events were found in 0/417 (0%) cGrade1, 19/270 (7.0%) cGrade2, 38/170 (22.4%) cGrade3, 32/73 (30.5%) cGrade4 and 47/102 (46.1%) cGrade5 patients. MFS is presented in Table 4 and Fig. 2b. Due to the absence of events in GG1 and cGrade1 tumours, for each grading system, the 1st and 2nd groups were clustered to enable statistical analysis. The continuous IQ-Gleason (*c*-index 0.834, 95%*CI* 0.802–0.863) and cGrade (*c*-index 0.834, 95%*CI* 0.808–0.866) both had comparable and highest discriminative value for MFS. The discriminative power of categorized IQ-Gleason (*c*-index 0.823, 95%*CI* 0.788–0.857) was also higher than that of conventional GG (*c*-index 0.806, 95%*CI* 0.777–0.839) (Fig. 3b).

**Table 4:** Cox proportional hazard models for metastasis-free survival stratified by grade group (GG), categorical integrated quantified Gleason (IQ-Gleason) score and cribriform grade (cGrade)

Group	Grade Group	IQ-Gleason	cGrade
	*HR (95%CI)*	*HR (95%CI)*	*HR (95%CI)*
1 & 2	*ref*	*ref*	*ref*
3	12.7 (7.2–22.4)	9.1 (5.2–16.2)	9.9 (5.7–17.2)
4	11.5 (6.5–20.4)	13.5 (8.1–22.6)	13.3 (7.5–23.6)
5	22.5 (13.0–39.0)	22.8 (13.2–39.1)	26.3 (15.3–45.2)

Groups 1 and 2 added together to avert eternity. All *p*-values are < 0.001

*CI* confidence interval, *HR* hazard ratio, *ref* reference

## Discussion

Gleason pattern 4 percentage, presence of IC/IDC and minor/tertiary Gleason patterns have been well acknowledged as independent prognostic features of prostate cancer. Therefore, according to the latest ISUP and GUPS recommendations, these pathological factors should be included in pathology reports in conjunction with the GS and GG [4, 5]. Albeit of clinical significance, it is yet unclear how to combine this pathological information into comprehensive risk stratification models for individual patients. Recently, two proof-of-principle studies have shown that the discriminative value of conventional GGs can significantly be improved by incorporating novel pathological characteristics in alternative grading systems [14, 18]. In this study, we show that continuous IQ-Gleason and cGrade both outperformed GGs particularly in prediction of MFS and to a lesser extent of BCRFS. These findings demonstrate that prostate cancer grading can significantly be improved by incorporating Gleason pattern 4 percentage, tertiary patterns and IC/IDC in new grading schemes.

This is the first study to independently validate IQ-Gleason for prediction of BCRFS after RP [14]. Furthermore, we show that the additive value of IQ-Gleason is even stronger for predicting MFS, which to our knowledge has not been reported yet. For comparison purposes, we also analysed IQ-Gleason as a 5-tier system, which outperformed GG for MFS but not for BCRFS, indicating that categorization led to significant loss of discriminative power. Finally, we confirm the findings of our previous biopsy study showing that cGrade has better discriminative ability for predicting MFS [18]. Both alternative grading systems led to considerable reclassification of original GGs, with only 51% of IQ-Gleason and 71% of cGrade categories being similar to the respective GG. For both alternative grading systems, the most prominent effect was the re-categorization of many GG2 patients with low-risk features as IQ-Gleason1 or cGrade1, respectively, doubling the number of men in the lowest risk category. While these men remained at very low risk of less than 1% for developing post-operative metastasis, BCR rates in IQ-Gleason1 and cGrade1 were higher than in GG1.

The GS/GG has been the global standard for prostate cancer grading for many years. Disadvantages of the current grading system are that it is prone to inter-observer variability and does not implement the new prognostic factors. The better discriminative value of cGrade is mostly related to the classification of the large group of GG2 men without IC/IDC as cGrade1. As cGrade is based on the GG system, it will still suffer from considerable inter-observer variability. At the same time, the IQ-Gleason assessment is time consuming and might result in delays in daily clinical practice. Yet, apart from its better performance, a strong point of continuous IQ-Gleason is that it is less susceptible to inter-observer variability related to the assessment of minor high-grade components. For instance, a RP with 70% GP3, 27% GP4 and 3% GP5 is graded as GG2 with tertiary pattern 5 according to ISUP, GUPS and WHO recommendations; however, if GP4 and GP5 quantities were assessed as 23% and 7%, respectively, GP5 would be regarded as a secondary component resulting in GG4. In both scenarios, the IQ-Gleason would, however, be 40 and remain unchanged. So, while subjective assessment can easily lead to significant alterations in GG categorization, the continuous IQ-Gleason score is more resistant to inter-observer variability.

In the 2019 ISUP survey on prostate cancer grading, the majority of respondents indicated they were open to altering the current GS/GG system by incorporation of new pathological parameters, but most felt more validation was needed before actually changing the current system [19]. While IQ-Gleason and cGrade both outperform current prostate cancer grading, we believe there is still room for further optimization. To determine how pathological factors should be weighed in such a system, it is important to determine the mutual interaction and collinearity of the variables. Most additional factors have been investigated as a single variable without including the other relevant covariates. In a GG2 biopsy cohort including both IC/IDC and Gleason pattern 4 quantity, Kweldam et al. showed that IC/IDC occurred more frequently with incremental Gleason 4 percentage, and that IC/IDC was the only independent predictive factor for post-operative BCRFS [11]. Similarly, Seyrek et al. found that GG2 RP specimens with a higher percentage of pattern 4 had more frequent IC/IDC and tertiary pattern 5, but that IC/IDC was the only independent factor for BCRFS [16]. Further study on the interaction of these pathological factors by other groups is required to identify their independent contribution to prostate cancer outcome.

Prostate cancer grading on biopsies is an important factor for therapeutic decision-making. Alternative grading systems could have added value in comprehensive risk stratification after biopsy, for instance supporting identification of candidates for active surveillance among IQ-Gleason1 and cGrade1 patients. While tumour grading at radical prostatectomy is mostly prognostic, the new grading schemes could have impact on patient communication and follow-up in the large group of IQ-Gleason1 and cGrade1 men.

The strong points of this study were the detailed monitoring of Gleason pattern percentages and growth patterns. The retrospective design, short median follow-up period of 54 months and relatively small sample size were restrictions, limiting the power of the statistical analyses. Furthermore, the current RP cohort was specifically enriched for high-grade tumours from other centres to increase statistical power in high-grade patients, which might have introduced a bias.

In conclusion, this is the first study validating the clinical performance of two alternative prostate cancer grading systems. We show that both IQ-Gleason and cGrade outperformed GGs in having better discriminative ability for MFS and BCRFS. This study shows that improvement of current prostate cancer grading is possible and could result in comprehensive incorporation of new prognostic pathological parameters in clinical practice.
